# The roles of the inhibitory autophagy regulator Rubicon in the heart: A new therapeutic target to prevent cardiac cell death

**DOI:** 10.1038/s12276-021-00600-3

**Published:** 2021-04-14

**Authors:** Jihoon Nah, Daniela Zablocki, Junichi Sadoshima

**Affiliations:** Department of Cell Biology and Molecular Medicine, Cardiovascular Research Institute, Rutgers New Jersey Medical School, Newark, NJ USA

**Keywords:** Macroautophagy, Mechanisms of disease

## Abstract

Autophagy contributes to the maintenance of cardiac homeostasis. The level of autophagy is dynamically altered in heart disease. Although autophagy is a promising therapeutic target, only a few selective autophagy activator candidates have been reported thus far. Rubicon is one of the few endogenous negative regulators of autophagy and a potential target for autophagy-inducing therapeutics. Rubicon was initially identified as a component of the Class III PI3K complex, and it has multiple functions, not only in canonical autophagy but also in endosomal trafficking and inflammatory responses. This review summarizes the molecular action of Rubicon in canonical and noncanonical autophagy. We discuss the roles of Rubicon in cardiac stress and the therapeutic potential of Rubicon in cardiac diseases through its modulation of autophagy.

## Heart disease: Preventing the death of heart muscle cells

A protein known as Rubicon, which inhibits the routine degradation of cell components in a process called autophagy, has been identified as a possible target for drugs to prevent the death of heart muscle cells. Autophagy is required for healthy cell maintenance and its failure has been linked to heart disease, cancer, metabolic disorder and aging. Jihoon Nah and colleagues at Rutgers New Jersey Medical School, Newark, USA, review the role of Rubicon in health and disease. The authors discuss how Rubicon suppresses autophagy in ways that could promote cell death and disease, focusing especially on the heart. They also identify significant areas where understanding of Rubicon’s regulatory effects is lacking. Some molecules that interfere with Rubicon’s activity have already been identified. Further research could yield new treatments for heart disease and other conditions.

## Introduction

Degradation of cellular components and organelles through autophagy helps to maintain cellular homeostasis. Autophagy is modulated during cellular stress to delay the onset of various diseases, including heart diseases. Failed autophagy has been linked to aging, cancer, metabolic disorders, and heart disease^1^. Therefore, maintaining autophagy function is an important goal for the development of therapeutics. Autophagy is tightly regulated by more than 35 kinds of autophagy-related (Atg) proteins. Although most Atg proteins positively regulate autophagy, together with Bcl-2, another autophagy regulator, Rubicon is one of the few known endogenous negative regulators of autophagy^2,3^.

In 2009, two groups identified Rubicon as a protein that suppresses autophagy by interacting with the Beclin 1 complex^2,4^. Rubicon inhibits autophagy and endosomal trafficking by directly interacting with Rab7^5^. On the other hand, Rubicon positively regulates LC3-associated phagocytosis and endocytosis, noncanonical forms of autophagy, in response to extracellular molecule stimuli^4,6^. Rubicon may be involved in disease progression through its effects on canonical or noncanonical autophagy (Table 1). Recent studies have identified the involvement of Rubicon in heart diseases^7,8^. Here, we review the current understanding of Rubicon function in canonical and noncanonical autophagy and summarize the roles of Rubicon in the heart. We also discuss the possible role of Rubicon as a new therapeutic target for various cardiac diseases.

**Table 1:** Phenotype of Rubicon-deficient mouse models.

Rubicon model	Stress	Phenotype	Ref.
Systemic knockout	Aging	Activates basal autophagy, reduces age-associated features	^48^
	LPS injection	Protects against lethality and contributes to maintaining cardiac stroke volume	^45^
Neuronal knockout (*Nestin-cre*)	α-Syn fibrils	Suppresses expansion of α-synuclein pathology	^48^
Hepatocyte knockout (*Alb-cre*)	HFD (high fat diet)	Ameliorates liver steatosis	^50^
Cardiomyocyte knockout (*Myh6-cre*)	I/R	Attenuates I/R injury and reduces autosis	^8^
Adipocyte knockout (*Adipoq-cre*)	Aging	Promotes metabolic disorder due to excess autophagy	^49^

### Rubicon in canonical autophagy

Rubicon is involved in multiple cellular functions, including autophagy, endosomal trafficking, phagocytosis, and inflammatory responses.

Autophagy is a highly conserved catabolic process in eukaryotes orchestrated by more than 35 Atg proteins that were first identified in yeast^3^. Cellular components are engulfed into a double-membrane structure, the autophagosome, in either a nonselective or selective manner, and the cargos are digested after fusion of autophagosomes with lysosomes^9^. Autophagy is initiated by the activation of an energy sensor, AMP-activated kinase (AMPK), which in turn inhibits mammalian target of rapamycin complex 1 (mTORC1) expression. This inhibition is followed by the activation of the Unc-51-like kinase 1 (Ulk1)/Atg1 complex, consisting of Ulk1, Atg13, FIP200, and Atg101^10^. Activated Ulk1/Atg1 directly phosphorylates AMBRA1, a Beclin 1-interacting protein, inducing translocation of the class III phosphatidylinositol 3 kinase (PI3K) complex to the endoplasmic reticulum (ER), where it initiates autophagosome formation^11^. The PI3K complex is composed of vascular protein sorting (VPS) 34, a catalytic subunit, Beclin 1, a scaffold protein, and VPS15, along with several additional interactors to initiate autophagosome nucleation. The PI3K complex also contains either Atg14L or UV radiation resistance-associated gene (UVRAG). The PI3K-Atg14L complex is localized at the ER, endosome, and isolation membrane and is essential for autophagosome formation, whereas the PI3K-UVRAG complex is localized primarily in the late endosome, thereby mediating endosomal trafficking^12^. It is unclear whether UVRAG-associated PI3K can control the maturation of autophagosomes. UVRAG plays a role in endosomal flux of cargo into lysosomes and autophagosome maturation by assembling class C type VPSs *without* Beclin 1/VPS34^13^.

**Fig. 1:**
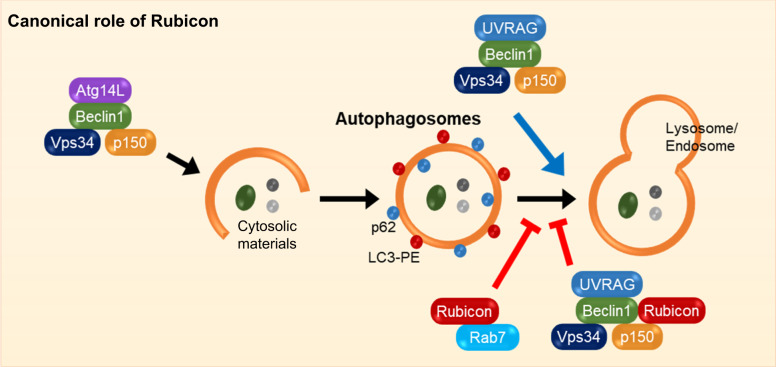
Role of Rubicon in canonical autophagy. Under autophagy-inducing conditions, the PI3KC3-Atg14L complex induces autophagosome formation and PI3KC3-UVRAG mediates autophagosome maturation or endosome trafficking. Autophagosome maturation or endosome trafficking is inhibited by Rubicon.

Rubicon was first identified as a Beclin 1-interacting protein. Rubicon regulates both the initiation of autophagy and autophagosome maturation^2^. Rubicon directly binds to the PI3K-UVRAG complex through its RUN domain and contributes to the suppression of autophagy initiation by inhibiting PI3K activity^14^. In addition, Rubicon interacts with UVRAG to inhibit lysosomal fusion with autophagosomes and endosomes. UVRAG stimulates lysosomal fusion of autophagosomes and endosomes by binding to the homotypic fusion and vacuole protein sorting (HOPS) complex^5^. Phosphorylation of UVRAG at Ser498 by mTORC1 enhances the UVRAG–Rubicon interaction, where Rubicon acts as an antagonist of UVRAG/HOPS-mediated autophagosome and endosome maturation^15^. On the other hand, Pacer, a protein associated with UVRAG that acts as an autophagy enhancer, antagonizes Rubicon to stimulate VPS34 kinase activity^16^. Rubicon is also known to be an endosomal trafficking mediator. Similar to PLEKHM1 and Pacer, Rubicon contains a Rubicon homology (RH) domain in the C-terminus. The RH domains of Rubicon and PLEKHM1 are crucial for their interaction with the late endosome small GTPase Rab7^17^. In contrast to Rubicon, which can interact with both Rab7 and the Beclin 1/VPS34 complex, PLEKHM1 cannot interact with the Beclin 1/VPS34 complex. Therefore, PLEKHM1 suppresses endocytic transport but not autophagosome maturation^17^. Rubicon may competitively interact with Rab7 and UVRAG. GTP-bound active Rab7 competes with UVRAG in the UVRAG-Rubicon complex, releasing UVRAG to associate with the HOPS complex and stimulating endosome maturation. In contrast, the interaction between UVRAG and Rubicon blocks Rab7 activation^5^. Thus, Rubicon acts as a negative regulator of endosomal trafficking and autophagosome maturation. Overexpression of Rubicon causes the accumulation of autophagosomes and lysosomes and blocks endosomal transport. On the other hand, Rubicon-depleted cells show increased autophagic activity and endocytic degradation^2^. In addition, recent studies have described posttranslational modifications of Rubicon during autophagy regulation. Hormonally upregulated neu-associated kinase (HUNK) phosphorylates the N-terminus of Rubicon, an important region for the interaction with VPS34. HUNK-dependent phosphorylation of Rubicon inhibits Rubicon-mediated autophagy inhibition, thereby promoting autophagic flux^18^ (Fig. 1).

### Rubicon in noncanonical autophagy

#### LC3-associated phagocytosis (LAP)

**Fig. 2:**
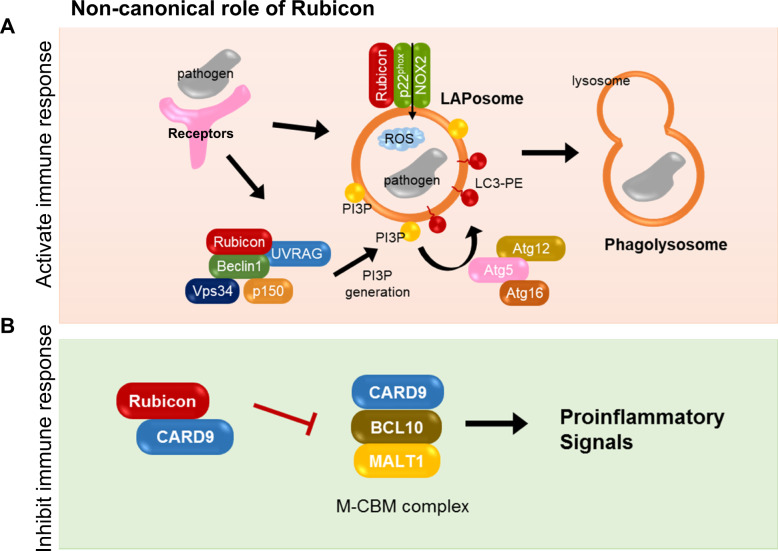
Role of Rubicon in non-canonical autophagy. **A** Recognition of extracellular pathogens activates the PI3KC3-UVRAG-Rubicon complex to generate PI3P on the engulfing phagosome. Rubicon-mediated PI3P generation stabilizes and activates the Rubicon-interacting NOX2 complex, thereby generating ROS. PI3P and ROS activate the Atg5-Atg12 conjugation system, thereby embedding LC3-PE in the phagosome membrane. LC3-embedded phagosomes (LAPosomes) fuse with lysosomes to degrade the engulfed pathogens. **B** Rubicon interacts with CARD9, thereby repressing the M-CMB complex, which is important for the generation of proinflammatory signals.

Recognition and uptake of extracellular molecules, such as dead cells, pathogens, or antigens, are essential cellular functions that prevent autoimmune or inflammatory responses. The autophagy machinery is involved in LAP, noncanonical autophagy triggered by several extracellular receptors, including Toll-like receptors (TLRs), Fc receptors (FcR), and TIM-4, a phosphatidylserine receptor^19^. These extracellular receptors trigger cellular signaling to recruit the core autophagy machinery, form a single membrane vesicle called a LAP-engaged phagosome (LAPosome), facilitate LAPosome maturation, and degrade cargo materials. LAP signaling is dependent on some of the classical autophagy mechanisms, including mechanisms involving Atg5, Atg7, Beclin 1, VPS34, UVRAG, and Rubicon. A key molecule for the formation of LAPosomes is lipidated LC3-II, which is embedded into the single membrane of LAPosomes^6^. Recognition of extracellular molecules activates the PI3K-UVRAG-Rubicon complex to generate PtdIns(3)P (PI3P) on LAPosomes. Rubicon-mediated PI3P generation on LAPosomes recruits the Atg5/7-dependent LC3 conjugation system and stimulates LC3-II embedment into the LAPosome membranes, which then triggers fusion of the LAPosomes with LAMP-1-containing lysosomes^20^. Interestingly, even though Rubicon blocks VPS34 kinase activity in the canonical autophagy process, it plays a crucial role in generating PI3P during LAP^20^. In addition, Rubicon-dependent PI3P generation on LAPosomes stabilizes and activates the nicotinamide adenine dinucleotide phosphate (NADPH) oxidase-2 (NOX2) complex to produce reactive oxygen species (ROS). Rubicon also transiently interacts with p22phox, a component of the NOX complex, to induce ROS generation^21^. ROS may not be essential for autophagosome formation in canonical autophagy, but they are critical for the progression of LAP^6^. NOX2-mediated ROS generation is critical for LAP maturation and phagosomal pH regulation^22^ (Fig. 2).

#### Rubicon in LC3-associated endocytosis (LANDO)

Recently, the involvement of a novel form of noncanonical autophagy that is facilitated by autophagy proteins, including Atg5, LC3, Atg16L, and Rubicon, was identified during endocytosis of amyloid beta (Aβ) in a mouse model of Alzheimer’s disease (AD). Aβ-containing endosomes are processed by LC3-associated endocytosis (LANDO). LANDO is essential for alleviating Aβ-induced inflammatory responses. Mice in which autophagy is maintained but that are deficient in LANDO exhibit a drastic increase in the production of proinflammatory cytokines, including TNFα, IL-1β, and IL-6, and the accumulation of neurotoxic Aβ in the brain^23,24^. Rubicon and Atg16L are dispensable for canonical autophagy, but they are essential for LANDO. Rubicon may play an essential role in the recruitment of LC3-II to Aβ-containing endosomes to prevent Aβ-induced neurotoxicity. Either Rubicon- or Atg16L-deficiency was sufficient to produce proinflammatory responses in the AD model. Although Rubicon facilitates phagosome maturation and elimination of cargo in LAP, it is not involved in Aβ degradation or endosome maturation by lysosomal fusion in LANDO^23^. Accumulating experimental and clinical evidence suggests that Aβ peptides also cause cardiovascular disease. Vascular deposition of Aβ peptides is involved in vascular inflammation, atherosclerosis, myocardial dysfunction, and cardiovascular disease-related mortality^25^. Understanding the role of LANDO in Aβ deposition may lead to the development of novel therapeutic interventions for vascular and cardiac deposition of Aβ in older patients.

#### The involvement of Rubicon in the inflammatory response

Inflammatory responses are activated in response to bacterial or viral infection to protect the host. Upon infection, immune cells produce cytokines that are important for the inhibition of virus replication and the induction of adaptive immune responses^26^. Rubicon activates LAP by interacting with p22phox to generate ROS and embedding LC3-II into the LAPosome. The Rubicon-mediated LAP process robustly increases inflammatory cytokines in response to infection^21^. However, Rubicon is also involved in the inhibition of host immune responses. The assembly of caspase recruit domain-containing protein 9 (CARD9), BCL10, and MALT1 to form the M-CBM complex is critical for central proinflammatory signaling in innate immune responses^27^. Rubicon physically interacts with CARD9, thereby inactivating the M-CMB complex. However, the Rubicon-CARD9 interaction is negatively affected by phosphorylation of Rubicon at Ser248, which is important for the interaction between Rubicon and 14–3–3. CARD9 and 14–3–3 competitively interact with Rubicon, and Rubicon acts as a feedback inhibitor of the CARD9-mediated inflammatory response to slow excessive production of inflammatory cytokines^28^.

To facilitate replication, many viruses enter the host cell through an endocytic pathway. Once inside the host, viruses utilize various host systems to escape endosomal/autophagic elimination^29^. For example, autophagy is often activated in response to viral infection to eliminate external invaders. Ironically, however, some viruses also facilitate autophagy to evade elimination by lysosomal degradation. Recent studies have demonstrated that Rubicon plays a role in viral replication. Hepatitis B virus (HBV) infection elevates Rubicon expression. This upregulation, in turn, promotes viral replication because the N-terminal RUN domain in Rubicon interacts with NEMO, an essential NF-κB modulator, inhibiting downstream signaling of NEMO, including phosphorylation of TBK1 and IRF3, which are essential for antiviral interferon (IFN) signaling^30^. Similarly, hepatitis C virus (HCV) enhances Rubicon expression to facilitate viral replication. The HCV NS4B protein is sufficient to induce Rubicon and suppress autophagy maturation^31^. On the other hand, increased Rubicon levels lead to the activation of the intracellular innate immune response, including the type 1 IFN pathway^32^. Kaposi’s sarcoma-associated herpesvirus (KSHV) is another virus that evades the host defense system via inhibition of autophagy activity. The KSHV K7 protein interacts with Rubicon to inhibit the maturation step of autophagy and attenuate VPS34 enzymatic activity^33^. During viral replication, Rubicon is upregulated to allow the virus to escape autophagic degradation. Thus, inhibition of Rubicon might be beneficial for the prevention of viral infection. Since viral infection has been implicated as a cause of myocarditis^34^, Rubicon may be considered a novel target for myocarditis treatment.

### Critical roles of Rubicon in cardiac diseases

#### Role of Rubicon in ischemia/reperfusion (I/R)

Ischemia/reperfusion injury is the major mechanism of injury in the heart when patients suffer from myocardial infarction. Despite extensive investigation to mitigate the effects of I/R, medical intervention has been marginally effective in humans. Myocardial I/R regulates autophagy in a time-dependent manner. Autophagy is activated during myocardial ischemia due to the low energy and hypoxic conditions in the heart. Interestingly, autophagy is further activated during the early phase of reperfusion through the upregulation of Beclin 1 expression and ROS production^35^. However, the Rubicon level gradually increases during reperfusion, and autophagic flux is simultaneously inactivated in a time-dependent manner^8^. After 6 h of reperfusion, autophagic flux is inactivated below basal levels due to the increased level of Rubicon in the border region of ischemia. The autophagosome formation rate remains elevated under this condition; however, suppression of autophagosome maturation due to Rubicon upregulation induces marked accumulation of autophagic vacuoles and facilitates autophagy-dependent cell death, termed autosis. Autosis is characterized by unique morphological features, including an increase in the numbers of autophagosomes, autolysosomes, and empty vacuoles, a characteristic perinuclear space, condensed mitochondria, and the disappearance of intracellular organelles. It is also characterized by its sensitivity to cardiac glycoside treatment. Importantly, autosis is accompanied by a shortage of essential cytosolic membranes but not excessive degradation of the cargos in lysosomes. It is speculated that excessive production of autophagosomes in the presence of Rubicon prevents the degradation and recycling of autophagosomes by lysosomes; thus, stimulation of autophagosome formation in this condition may deplete endomembrane systems^9^. This hypothesis requires further experimentation for verification. Interestingly, cardiomyocyte-specific conditional knockout of Rubicon restores autophagic flux and reduces the autotic cell death rate during the late phase of reperfusion in the heart^8^. No effective treatment to reduce myocardial ischemia/reperfusion injury is currently available. Thus, targeting autosis through inhibition of Rubicon may be a unique and promising approach to reduce the extent of myocardial injury in patients with I/R injury.

CARD9 has been shown to be involved in several cardiac diseases, including coronary artery disease, cardiac fibrosis, heart failure and carotid atherosclerosis^36^. CARD9 protects cardiomyocytes during I/R injury by inhibiting apoptosis^37^. In addition, CARD9 also promotes autophagic activity through interaction with Rubicon. As discussed above, autophagic flux is inhibited during the late phase of reperfusion through upregulation of Rubicon. Overexpression of CARD9 reverses Rubicon-induced suppression of autophagic flux. Rubicon is translocated from the PI3K-UVRAG complex to CARD9, allowing an increase in the activity of VPS34. In fact, silencing CARD9 impairs autophagic flux, whereas overexpression of CARD9 promotes autophagy and protects the heart from reoxygenation injury^38^.

#### Role of Rubicon in doxorubicin-induced cardiotoxicity

Doxorubicin (DOX) is an effective chemotherapy for various types of cancer. However, detrimental side effects on the heart limit its clinical application^39^. DOX induces acute and chronic cardiotoxicity, and cardiac autophagy is dynamically regulated by DOX treatment. Recent studies have shown that DOX impairs the maturation step of autophagy and induces cardiotoxicity through suppression of autophagic flux^40^. Other researchers have demonstrated that excessive autophagy contributes to cardiotoxicity through autophagic cell death^41^. Thus, DOX treatment may induce excessive accumulation of autophagosomes. UVRAG is an important binding partner of Rubicon in the regulation of VPS34 activity. UVRAG deficiency aggravates impaired autophagic flux and elevated ROS generation in DOX-treated hearts and exacerbates acute and chronic DOX-induced cardiotoxicity^42^. On the other hand, the loss of Rubicon ameliorates DOX-induced cardiotoxicity. Similarly, autophagic flux and mitophagy activity after DOX treatment were improved in Rubicon-deficient hearts. These results suggest that Rubicon deficiency improves DOX-induced cardiotoxicity through enhancement of autophagic flux, which not only prevents excessive accumulation of autophagosomes but also improves cellular quality control mechanisms^7^.

#### Role of Rubicon in inflammation-induced cardiotoxicity

Cardiovascular dysfunction is one of the common complications of sepsis. In experimental animals, lipopolysaccharide (LPS) injection triggers systemic inflammation that mimics sepsis. LPS treatment induces a significant drop in LV stroke volume and cardiac output^43^. In addition, cardiac autophagy is dynamically changed during sepsis. Recent studies revealed that whereas low doses of LPS induce autophagy as an adaptive response, high doses of LPS impair autophagic flux. Thus, autophagy is activated as an adaptive response during the early phase of sepsis or mild sepsis, whereas autophagy is maladaptive during severe sepsis^44^. The mechanism through which autophagy is differentially regulated in the early and the late stages of sepsis is unknown. In mouse models, Rubicon deficiency enhances autophagic flux in the heart during LPS-induced sepsis, thereby maintaining cardiac stroke volume but not affecting myocardial inflammatory responses^45^.

#### Role of Rubicon in aging

The risk of heart disease increases dramatically with age^46^. Autophagy activity is gradually reduced with aging in several organisms. Age-dependent changes in the upstream mechanisms of autophagy may be involved in the aging-dependent decline in autophagy in the heart^47^. A recent study revealed that Rubicon is upregulated in aging at both the mRNA and protein levels in worms, flies, and mouse kidneys, which indicates that Rubicon may contribute to the aging-dependent impairment of autophagy. Interestingly, knockdown of Rubicon extends the lifespan of worms and flies and ameliorates several age-related phenotypes^48^. On the other hand, Rubicon is critical for maintaining adipocyte metabolism. In contrast to other tissue cells, in aged adipocytes, the level of Rubicon declines, and autophagic activity is excessively induced, thereby exacerbating metabolic disorders through degradation of SRC-1 and TIF2, coactivators of PPARγ^49^. Although aging is an important facilitator of cardiac disorders, it remains unknown whether cardiac Rubicon is upregulated in humans with aging; if it is upregulated, then it may contribute to age-dependent cardiac dysfunction.

### Rubicon as a new therapeutic target of cardiac diseases

Rubicon facilitates multiple cellular responses in addition to autophagy in a context-dependent manner. Under some conditions, such as LAP, LANDO, or inflammatory responses, Rubicon serves as an adaptive molecule, protecting cells during stress. However, Rubicon can also act as a maladaptive protein by inhibiting general autophagy. Upregulation of Rubicon accelerates apoptosis or autosis in a context-dependent manner^8,50^. Therefore, silencing Rubicon promotes cell survival. For example, inhibition of Rubicon may be salutary during aging and some cardiac conditions, including myocardial reperfusion, doxorubicin cardiomyopathy, and sepsis. We briefly discuss the structure of Rubicon and its relevance in the development of Rubicon modulators.

Rubicon is expressed in most tissues and organs, is mainly localized in endosomes or lysosomes and is composed of multifunctional domains the interact with multiple partners. Rubicon interacts with VPS34 via the RUN domain, UVRAG and Beclin 1 via the coiled-coil domain (CCD), p22phox via the serine-rich C-terminus (SR-C) domain, 14–3–3β via the serine-rich N-terminus (SR-N) domain, CARD9 via the helix-coil-rich (H-C) domain, and Rab7 via the FYVE-like motif^19,51^ (Table 2). Recently, the interaction between Rubicon and the PI3K complex III (PI3KC3)-UVRAG complex was validated by cryo-EM. The PI3KC3-UVRAG complex acts as a mediator of phagophore expansion and endosomal trafficking through its interaction with Rubicon^52^. PI3KC3 is connected to the cell membrane through the Beclin 1 BARA domain^53^. The middle domain of Rubicon, which includes the CCD, forms a stable complex with the PI3KC3-UVRAG complex, allowing Rubicon to inhibit PI3KC3 through its interaction with the Beclin 1 BARA domain^51^. Cryo-EM and HDX-MS analyses showed that of the 3 α-helices formed by residues 471–672 of Rubicon, the first, helix α1, is critical for the inhibition of PI3KC3-UVRAG activity. Specifically, the PI3KC3-UVRAG complex dissociates from the membrane through a conformational change in the Beclin 1 BARA aromatic docking region in the presence of Rubicon^51^. Interestingly, the sequence of the docking region of the BARA domain is very similar to the autophagy-activating peptide Tat-Beclin 1^54^. In contrast to Rubicon, Tat-Beclin 1 penetrates into the BARA domain and supports the expanded membrane docking of PI3KC3. Thus, the interaction between Rubicon and the Beclin 1 BARA domain may be a good target for triggering the activation of autophagy.

**Table 2:** Rubicon-interacting proteins.

Interactors	Binding region	Function of Rubicon	Ref.
VPS34	RUN domain (49–180 a.a.)	Inhibits VPS34 lipid kinase activity and suppresses autophagy	^14^
UVRAG	CCD domain (300–600 a.a.)	Suppresses autophagosome and endosome maturation	^14,15^
Beclin 1	CCD domain (505–557 a.a).	Inhibits VPS34 lipid kinase activity and suppresses autophagic flux	^21,51^
p22phox	SR-C domain (567–625 a.a.)	Mediates phagocytic NADPH oxidase activation in response to microbial infection	^21^
14–3–3β	SR-N domain (204–447 a.a.)	Competitively interacts with 14–3–3β and CARD9	^28^
CARD9	H-C domain (625–760 a.a.)	Prevents an unbalanced CARD9-BLC10-MALT1-mediated proinflammatory response	^28^
Rab7	FYVE-like motif (721–972 a.a.)	Negatively regulates the endocytic pathway and autophagosome maturation	^17,55^
NEMO	undefined	Inhibits type-I interferon production and enhances viral replication	^30^

**Fig. 3:**
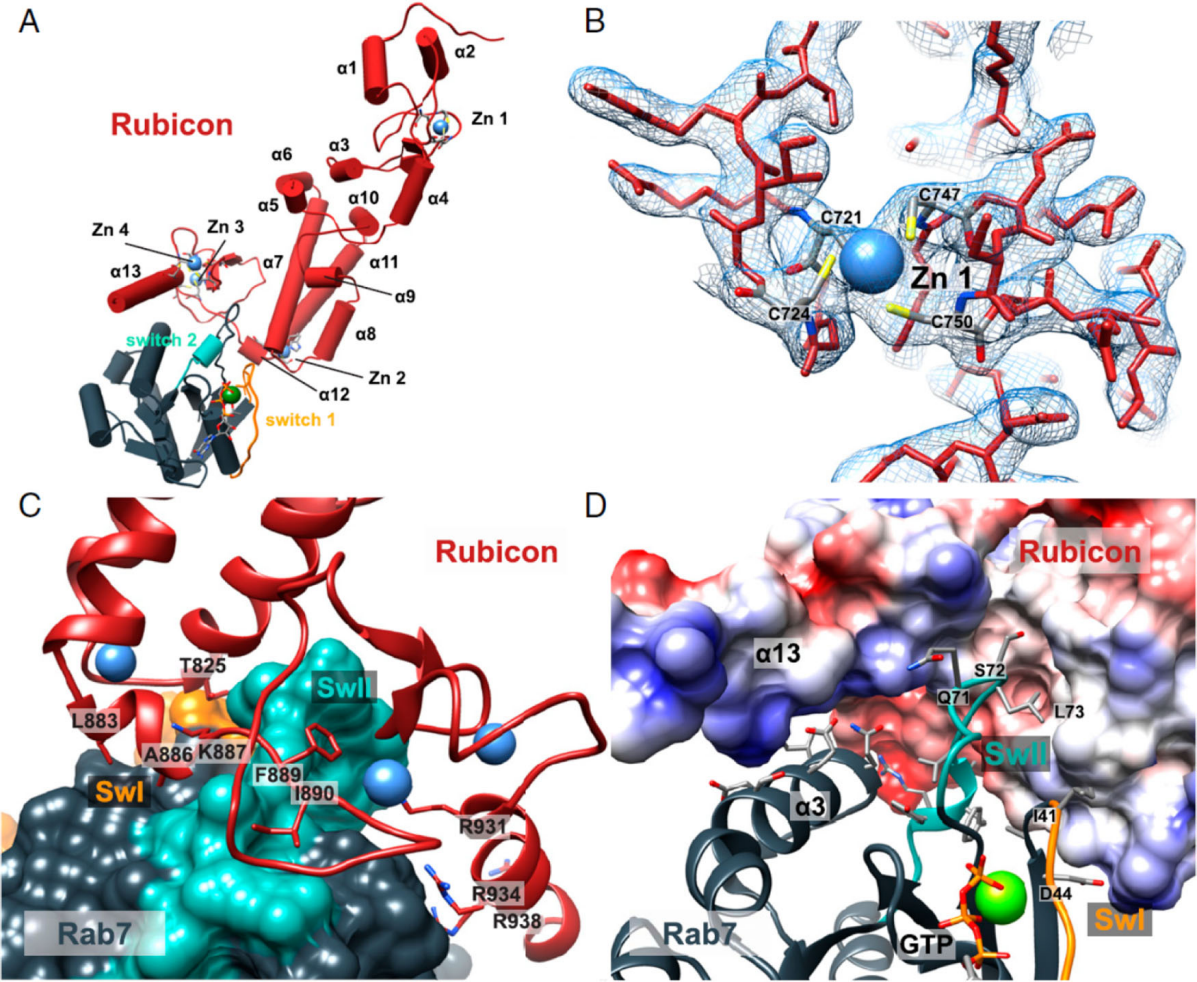
Structure of the interaction between Rubicon and Rab7. **A** Cylindrical representation of the Rubicon:Rab7 complex and defined zinc fingers. **B** The 2.8-Å 2Fo–Fc composite omit map on zinc finger 1. **C** Ribbon representation of the Rubicon RH domain, which is important for binding with Switch II region on Rab7. Key interacting Rubicon residues are described with their numbers. **D** Surface representation of human Rubicon colored by Coulombic potential and ribbon representation of Rab7 with key residues in the interaction described with their numbers^55^. Copyright (2020) National Academy of Sciences.

Rubicon also interacts with Rab7 through its C-terminal zinc cluster-containing RH domain^17^. Small GTPases, including Rab7, regulate intracellular signaling pathways by interacting with their binding partners. Two highly important loop regions, Switch I and Switch II, are regulated by guanine nucleotide hydrolysis and are involved in protein-protein interactions. The hydrophobic center of the Switch II region (72–77 a.a.) of Rab7 makes extensive contact with the Rubicon side chain at Met822 and Thr825 and the aliphatic parts of Asn821 and Lys887^55^ (Fig. 3). Since the interaction between Rubicon and Rab7 is essential for attenuation of autophagy maturation, interventions that interfere with this region may stimulate autophagic flux.

Rubicon has been proposed as a therapeutic target for septic shock-induced inflammatory responses. Rubicon interacts with p22phox upon microbial infection and induces inflammatory cytokine production. p22phox is a component of the NOX complex, which is critical for superoxide production. The rubicon-p22phox interaction plays a role in LAP, with ROS activating LAP in response to infection^20^. Since Rubicon interacts with an N-terminal 8-amino acid peptide of p22phox (N8 peptide), exogenous N8 peptide interferes with the Rubicon-p22phox interaction and suppresses ROS and inflammatory cytokine production. The tat-N8 peptide reduces the mortality associated with polymicrobial sepsis by inhibiting hyperactivation of the inflammatory system in mice^56^. An additional study identified 2-(tetrahydroindazolyl)phenoxy-N-(thiadiazolyl)propanamide 2 (TIPTP) as a potent and less toxic inhibitor of the Rubicon–p22phox interaction. Rheumatoid arthritis (RA) is a chronic autoimmune and inflammatory disease that causes joint pain and damage. Hypergeneration of ROS by the NOX complex is an important cause of the pathology of RA^57^. Interestingly, the Rubicon–p22phox interaction is increased during RA progression. TIPTP robustly suppresses the Rubicon–p22phox interaction and the ROS-mediated inflammatory response in RA^58^.

Currently, there is no modulator of Rubicon that selectively targets the canonical autophagy pathway. As discussed above, since silencing Rubicon potentially alleviates some cardiac conditions by normalizing or enhancing autophagic flux, developing novel inhibitors to disrupt the Rab7–Rubicon interaction is of great interest.

## Concluding remarks

Modulation of autophagy has the potential to treat or prevent cardiac diseases. To target autophagy for the treatment of cardiac diseases, it is necessary to understand how autophagy is regulated and the precise role of autophagy in a given cardiac condition. Among the multiple steps of autophagy, autophagosome-lysosome fusion is often modulated under stress conditions. However, the molecular mechanism controlling this step remains poorly understood. Rubicon is a key regulator of autophagy maturation, autophagosome-lysosome fusion, and endosomal trafficking. It is one of the few endogenous inhibitory regulators of autophagy and participates in noncanonical and canonical autophagy processes. Rubicon has recently been shown to play a causative role in promoting pathologies, including myocardial reperfusion injury. Thus, targeting Rubicon may be a promising modality of autophagy modulation in various cardiac conditions.
